# Determinants of healthcare seeking for childhood illnesses among caregivers of under-five children in urban slums in Malawi: a population-based cross-sectional study

**DOI:** 10.1186/s12887-020-1913-9

**Published:** 2020-01-17

**Authors:** Edgar Arnold Lungu, Catherine Darker, Regien Biesma

**Affiliations:** 1 UNICEF Zambia, Health and HIV Section, P.O Box 33610, Lusaka, Zambia; 2 Department of Public Health & Primary Care, Trinity College Dublin, Tallaght Hospital, Dublin 24, 24 Ireland; 3 Global Health Unit, Department of Health Sciences, University of Groningen, P.O Box 196, 9700 AD Groningen, Netherlands

**Keywords:** Urban slums, Healthcare seeking, Under-five children, Malawi

## Abstract

**Background:**

There is considerable evidence that health systems, in so far as they ensure access to healthcare, promote population health even independent of other determinants. Access to child health services remains integral to improving child health outcomes. Cognisant that improvements in child health have been unevenly distributed, it is imperative that health services and research focus on the disadvantaged groups. Children residing in urban slums are known to face a health disadvantage that is masked by the common view of an urban health advantage. Granted increasing urbanisation rates and proliferation of urban slums resulting from urban poverty, the health of under-five children in slums remains a public health imperative in Malawi. We explored determinants of healthcare-seeking from a biomedical health provider for childhood symptoms of fever, cough with fast breathing and diarrhoea in three urban slums of Lilongwe, Malawi.

**Methods:**

This was a population-based cross-sectional study involving 543 caregivers of under-five children. Data on childhood morbidity and healthcare seeking in three months period were collected using face-to-face interviews guided by a validated questionnaire. Data were entered in CS-Pro 5.0 and analysed in SPSS version 20 using descriptive statistics and logistic regression analyses.

**Results:**

61% of caregivers sought healthcare albeit 53% of them sought healthcare late. Public health facilities constituted the most frequently used health providers. Healthcare was more likely to be sought: for younger than older under-five children (*AOR = 0.54; 95% CI: 0.30–0.99)*; when illness was perceived to be severe (*AOR = 2.40; 95% CI: 1.34–4.30)*; when the presenting symptom was fever (*AOR = 1.77; 95% CI: 1.10–2.86).* Home management of childhood illness was negatively associated with care-seeking (*AOR = 0.54; 95% CI: 0.36–0.81*) and timely care-seeking (*AOR = 0.44; 95% CI: 0.2–0.74)*. Caregivers with good knowledge of child danger signs were less likely to seek care timely (*AOR = 0.57; 95% CI: 0.33–0.99).*

**Conclusions:**

Even in the context of geographical proximity to healthcare services, caregivers in urban slums may not seek healthcare or when they do so the majority may not undertake timely healthcare care seeking. Factors related to the child, the type of illness, and the caregiver are central to the healthcare decision making dynamics. Improving access to under-five child health services therefore requires considering multiple factors.

## Background

Global trends pertaining to child health show a significant decline in child morbidity and mortality over the past few decades reflecting successes of various public health efforts [1–3]. Malawi provides a specific example of this improvement granted that it achieved Millennium Development Goal (MDG) 4 which demanded reducing child mortality by two thirds by 2015, from the 1990 levels [4]. Specifically, the MDG 4 target for Malawi was an under-five mortality rate of 76 deaths per 1000 live births [5] but the country surpassed this target, registering an under-five mortality rate of 63 deaths per 1000 live births [6]. This represents a remarkable 72% reduction in under-five mortality rate from the 227 deaths per 1000 live births estimated for 1990 [5, 7]. It is noteworthy that such a significant achievement in child health has occurred in the context of a health system with multiple constraints including; inadequate health workforce, limited health infrastructure, equipment and essential supplies, and consistently low levels of health financing [8, 9].

The substantial achievements notwithstanding, child health indicators for Malawi, such as under-five mortality rate of 63 deaths per 1000 live births, neonatal and infant mortality rates of 27 and 42 deaths per 1000 live births respectively [6] remain causes for concern. Promoting access to proven cost-effective child health interventions is central to child mortality reduction [10]. Evidently, Walker and others projected that under-five mortality would reduce from the estimated 7.6 million in 2010 to 2.3 million by 2035 if each country accelerated coverage of child health interventions to the highest level attained by a country with similar levels in 2010 [11]. The Integrated Management of Childhood Illnesses (IMCI) represents the main approach to promoting access to healthcare across a continuum of care including a community and health system focus through its three interconnected components namely: (i) clinical/facility IMCI (provision of holistic clinical care to sick children); (ii) community IMCI (promoting good family practices for child care including healthcare seeking practices, basic treatment by lay cadres) and (iii) health systems strengthening (provision of skills, supplies and equipment to support quality child health service delivery). IMCI has been implemented in Malawi since the late 1990’s and is lauded for its contribution to increasing coverage of essential interventions and consequent significant improvements in child health [8].

Increased access to essential child health services therefore remains integral to efforts to achieve Sustainable Development Goals (SDG) particularly target 3.1 that asserts “End preventable deaths of new-borns and children under 5 years of age, with all countries aiming to reduce neonatal mortality to at least as low as 12 per 1,000 live births and under-5 mortality to at least as low as 25 per 1,000 live births” [12].

The principle of leaving no one behind, entailing universal health coverage is central to the SDG agenda. However, it is recognised that improvements in child health have been unevenly distributed within and across countries [1, 13, 14]. Rural-urban differentials in child health indicators are a case in point. For example, Malawi demographic and health survey indicate an under-five mortality rate of 77 deaths per 1000 live births in rural compared to 60 deaths per 1000 live births in urban areas [6]. Justifiably, ensuring that health services reach the rural population has been one of the policy priorities of Malawi’s Ministry of Health [15–17]. However, evidence is abound suggesting that certain urban population groups, such as the urban poor living in urban slums, characterised by overcrowding; inadequate access to safe water; sanitation; insecurity of tenure; poor housing and inadequate public services experience a health disadvantage [18–25].

The urban slum environment impacts on child health [18, 21, 24, 26] particularly posing risk to vulnerable population groups such as children. Evidence from Kenya suggests that infant and child mortality rates in urban slums of Kibera and Embakasi were three to four times higher than the Nairobi average and higher than the rural average [26]. This phenomenon has also been reported in other settings including Bangladesh and Egypt [27] and other developing country contexts [23]. In Malawi, the trend of a declining urban advantage noted for some child health indicators is arguably due to relatively poor indicators among urban poor mostly residing in slum conditions pulling down aggregate measures for urban when compared to rural setting [28].

Evidence from elsewhere suggests that limited access to health services prevail to the disadvantage of the poorest households [29] despite geographical access to health facilities and free services at the point of use [30, 31]. Nevertheless, appropriate disaggregated and/or specific information pertaining to some vulnerable urban populations such as those residing in urban slums is presently lacking in Malawi and other countries [22, 28, 32]. As Garenne posited, large demographic and health surveys hardly focus on the urban poor and slum areas hence not providing the much needed information on an important area of urban health [33]. Moreover, the implications of urbanization on child health outcomes and service coverage have been documented [24]. For Malawi, being among the least urbanized but also one of those with fastest urbanization rates, such as 6.2% for Lilongwe the capital city [34], understanding and planning for adequate access to child health services by caregivers in urban slums should be a public health imperative.

Some few studies conducted on healthcare seeking for under-five children in urban slums have found that while care-seeking may not be that low, it is of questionable quality and that poverty represents the most significant impediment to accessing health services [35, 36]. Health system factors including attitude of health workers, distance to health facility, thoroughness of health examination, availability of medicines and supplies, cost of healthcare have been cited to influence care-seeking decisions for sick children among the urban poor and slum residents in Malawi [37, 38]. These studies have largely focused on supply side factors and only used qualitative methods.

We investigated factors that determined child healthcare seeking among caregivers for three common childhood symptoms of fever, cough with fast breathing, and diarrhea in three urban slums in Lilongwe, Malawi. Our research contributes to literature in understanding the dynamics of child healthcare seeking among caregivers of under-five children focusing on demand side factors in the context of an urban slum in a developing country. We view our study as being potentially useful in contributing to efforts to promote child health, survival and development, granted the context of increasing urbanization and the dearth of evidence among urban slum populations.

## Methods

### Design

This study used a cross-sectional design to determine factors associated with seeking Under-five child healthcare services. It was part of a larger longitudinal study on child health and survival in urban slums conducted between October 2011 and February, 2013 in Lilongwe.

### Setting

The study was conducted in three urban slums of Senti, Mgona and Ntandire in Lilongwe, Malawi’s capital city. Given that the geographical focus of the study was in slum setting, the three study sites were chosen as they met the classification of an urban slum (i.e. overcrowding; inadequate access to safe water; sanitation; insecurity of tenure; poor housing and inadequate public services). Details on selection of the three study sites are described in detail elsewhere [37] but entailed two key steps namely: we identified all areas befitting the slum classification in Lilongwe City; and then undertook a simple random selection to identify the three as study sites.

Lilongwe, with a projected population of 1.9 million in 2017, of which about 675,000 reside in the city [39] is the fastest urbanising of Malawian cities and has some of the worst child health indicators. The recent Malawi Demographic and Health Survey 2015/16 reported district specific Under-five child mortality rate for Lilongwe to be 84 deaths per 1000 live births which is the fifth highest of the twenty eight (28) districts and much higher than national level of 63 deaths per 1000 live births [6].

None of the three sites have a public health facility within its geographical boundaries. At the time of data collection, there was one private health facility within Senti, none in Mgona and Ntandire although a facility run by a faith based institution is located just outside Ntandire and closer than the public health facility that is mostly utilized by Ntandire residents. All three areas have diverse ethnic groups, a reflection of heterogenous urban populations.

### Participants selection

To select participants for the study, we used an eligibility criteria that constituted: child below five years of age (and caregiver), residence in the study setting for not less than six months, provide consent to participate, having no intentions of migrating granted the requirement for longitudinal observation of the larger study.

We undertook our own household census through a house-to-house community walk through to enumerate eligible study participants. Due to high likelihood of population mobility associated with urban slums and the fact that the household listings from the 2008 Population and Housing Census were mostly outdated (denoting under-five children in 2012 from 2008 data) for our purpose, we decided to undertake census for this study. Households which may have experienced a recent death of an under-five child were not included in this enumeration (unless they also had a living child under the age of 5 years) given that the larger study design (prospective cohort) entailed prospective follow up of healthcare seeking for childhood morbidities. A total of 2860 pairs of under-five children and caregivers were enumerated in all three areas with the following distribution: 1464 in Ntandire, 922 in Mgona and 474 in Senti.

### Sample size and sampling

We estimated a sample size using the formula: *n* = (*z*)^2^ × *p*(1 − *p*)/*e*^2^ where *n* is the sample size, ***z*** is the value at 95% Confidence Interval (= 1.96), ***e*** is the standard error (estimated at 0.1) and ***p*** is the probable rate of appropriate care seeking. Based on literature, we assumed that 50% of our study participants reporting morbidity with any of the symptoms of interest would seek care from a biomedical health provider. Indeed, evidence from literature such as Malawi Demographic and Health Survey 2016 reported 59% of care givers from urban setting seeking health care for their children with fever symptom (same rate of healthcare seeking for diarrhea) and 50% of care seeking from a biomedical health provider for children with fever as reported by an IMCI key practices follow up study.

Using this formula, a minimum sample size of 384 was arrived at. Granted that this was part of a longitudinal study for which participant attrition is a risk [40] and the fact that urban slum populations are known for their mobility [41] we factored an increase of about 40% to the calculated sample size. Ultimately the study sample size was 543 pairs of under-five children and caregivers.

Probability Proportionate to Size (PPS) sampling was used to denote area specific contribution to the overall study sample size of 543 leading to: 245; 188 and 110 caregivers of Under-five children from Ntandire, Mgona and Senti respectively. A simple random selection using computer generated numbers from Microsoft Excel was conducted to select participants for the study in each of the study settings as per PPS sample size calculation. We undertook this by following a three steps process: (i) eligible participants enumerated from the census were given numerical codes within their slum setting, meaning Ntandire had numerical codes from 1 to 1464, Mgona and Senti’s codes were 1 to 922 and 1 to 474 respectively; (ii) Using Microsoft Excel, we generated random numbers commensurate with sample size of respective setting, for example, 245 numbers were generated randomly between 1 to 922 for Mgona; (iii) the generated random numbers were then matched with numerical codes in the database of eligible participants from the census for respective slum settings to select participants for the study.

### Data collection

Face to face interviews in local Chichewa language were conducted by research assistants who had undergone training in the study protocol and interviewing techniques. Guided by a structured questionnaire, research assistants asked care-givers questions about their Under-five child. Interviews were conducted in participants’ homes using a structured questionnaire that took about 45 to 60 min to administer. The questionnaire was based on the validated data collection tool for the Demographic and Health Survey (DHS) and a follow up survey for key family practices for Community Integrated Management of Childhood Illnesses (C-IMCI) in Malawi. Both the DHS and Follow up survey on C-IMCI practices questionnaires are robust, have previously been pretested, are widely accepted and their internal validity has been proven. The questionnaire largely constituted information on: sociodemographic characteristics, childhood illnesses, healthcare seeking, and knowledge of child danger signs, other key IMCI family practices and health service availability. Information on childhood morbidity and care-seeking from a health facility were also objectively verified from child health passports.

### Data quality assurance

Training of research assistants on the study protocol including eligibility criteria, data collection instruments, and in interviewing techniques emphasized the need for adherence to protocol and maintaining data quality. Field supervisors conducted spot checks and immediate review of questionnaires upon being submitted by data collectors to ensure consistency and completeness of the data collected. The questionnaire was also pre-tested on 30 people prior to main data collection.

### Data management and analysis

#### Variable definition

The dependent variables were: care-seeking for childhood illness defined as seeking healthcare from any biomedical health provider; and timely care-seeking described as healthcare sought within 24 h from onset of childhood illness symptoms. The basis for this time reference being the commitment that heads of states made at the Roll Back Malaria (RBM) summit of 2000 in Abuja that those suffering from malaria should have access to appropriate, affordable and correct treatment within 24 h of symptom onset [42]. For the same reason that prompt access to effective health care is essential not only for malaria, this definition was applied to ARI and diarrhea being other child illness symptoms of interest in this study. However, given the challenges with comprehending 24 h in the context of seldom use of watches for hour estimations and also high illiteracy levels, timely care seeking essentially entailed ‘by the next day’ from the point symptoms had begun or a symptom definition had been met (in the case of diarrhea as will be explained subsequently).

Independent variables for the study included: age of child, age of caregiver, slum residence, caregiver’s autonomy, caregiver’s social capital, household wealth, type of childhood morbidity (symptoms), knowledge of child danger signs, perceived severity of child illness, and home management of childhood illness. We subsequently provide operational definitions for some independent variables that may not be obvious.

##### Child morbidity/symptom definition

Child illness symptoms were defined according to the IMCI classification and were explained for easy comprehension by caregivers as follows:

##### Fever

If caregiver reported that during the illness episode in question, the child’s body felt hot (higher than normal temperature as perceived/felt by caregiver) or body temperature was taken and it was 37.5 degrees Celsius or above).

##### Cough with fast breathing

If caregiver reported that child was coughing and according to him/her also had faster breaths than usual or if caregiver generally described coughing with difficulty breathing.

##### Diarrhoea

If caregiver reported that child had three or more loose stools in a period of 24 h. Given difficulties with estimation of 24 h, data collectors usually probed before classification and the general description of 24 h typically entailed ‘for the whole day from morning to night’ or ‘started later in the day but continued into the night’ or ‘started in the night and continued to next morning’.

##### Caregiver’s autonomy

Autonomy referred to ability to make independent decisions or to have the right to contribute to a decision in a family setting. Questions on autonomy were adapted from a standard scale used by Fantahun and others in Ethiopia [43] which were deemed relevant for the setting. Autonomy score was recoded based on responses to four questions pertaining to decision making powers such as: who makes ‘big decisions’ (decisions to change place of residence, buy, sell or reconstruct a house, rent land, etc.); routine household decisions that include decisions on buying and selling food items and day-to-day activities in the household; decisions to visit family and friends; and decisions to take a sick family member to a health facility. A code of 0 was attached to decision making that did not involve caregiver and 1 where caregiver was either involved or was the sole decision maker. An aggregate score of 0–2 was classified as low autonomy and 3–4 as high autonomy of caregiver.

##### Caregiver’s social capital

This broadly entails behavior manifestations of social connections and reflects attitudes such as trust in others and norms of reciprocity with people. Social capital was recoded from responses to five questions on the questionnaire adapted from a tool used by Fantahun and others [43] which included: ability to borrow money in case of need, membership and/or leadership of social support groups, membership of community organisations, trusting people, thinking that people can hurt. A coding of 1 was given if present and 0 if absent for all questions except ‘people can hurt’ where a reverse coding was applied during data collection. A social capital variable was recoded with scores of 0–2 and 3–5 computed for low and high social capital scores respectively.

##### Household wealth

Household wealth was constructed using Principal Component Analysis (PCA) in SPSS based on asset indices from asset questions adapted from the demographic and health surveys questionnaire. The information used emerged from questions on: housing structure (roof, floor, and wall materials); type of toilet; source of energy for cooking; ownership of radio, bicycle, refrigerator etc. Based on the Eigen values from the sample, household wealth categories were constructed and categorised into low, medium and high which corresponded to three equal categorisation of the maximum Eigen value. This approach was adopted from construction of household wealth indices in Demographic and Health Surveys which is widely accepted as a measure of household socioeconomic position [6, 44].

##### Knowledge of child danger signs

The IMCI strategy regards recognition of danger signs as central to care-seeking hence its implementation requires educating caregivers on child danger signs. In our study, caregivers were asked to mention childhood danger signs that warrant immediate care-seeking from a health facility. The unprompted responses were measured against a list of seven general child danger signs on the questionnaire as stipulated by IMCI. These danger signs commonly result from the three conditions/symptoms (fever, ARI and diarrhoea) and include that a child is: vomiting everything ingested, unable to drink or breastfeed, unconscious, extremely weak, convulsing, passing little and dark urine, and has sunken eyes. A dichotomous variable was computed where ‘no/very little knowledge’ represented zero to knowledge of up to two child danger signs and ‘knowledgeable’ entailed knowledge of three or more of the six danger signs in IMCI.

### Statistical analysis

Data were entered into CSPro 5.0, checked for consistency, completeness and accuracy then transferred to and analysed with SPSS version 20 for Windows. Descriptive analysis of healthcare seeking and timely care-seeking were conducted for the aggregate sample and according to different child and caregiver characteristics. Multivariate logistic regression analysis was used to identify factors that determined care-seeking and timely care-seeking for under-five childhood morbidity. Only variables that were significant at 0.1 in univariate logistic regression were included in the final multivariate models.

### Ethical approval

The study protocol was approved by the National Health Sciences Research and Ethics Committee in Malawi and the Health Policy and Management-Centre for Global Health Research and Ethics Committee of Trinity College Dublin, Ireland. Participants also provided informed verbal or written consent depending on literacy levels, prior to data collection. Community/ward leaders were consulted and provided permission for community entry prior to data collection. Access to data was restricted to members of the research team.

## Results

### Sociodemographic characteristics of participants

The study had a high acceptance rate (99.6%) as almost all (543 of the 545) eligible participants who were approached to participate in our study responded to the questionnaire. The two (0.4%) who declined to participate indicated they needed consent from the spouse (husbands) but did not return. The majority of respondents were female (99%) and married (89%) (See Table 1). The predominance of females as primary caregivers is consistent with socio-cultural norms in the study context. While the Chewa and Ngoni ethnic groups were in a small majority (31 and 28% respectively), the ethnicity distribution reflects the heterogeneity of urban areas. The mean age of caregivers was 28 years (SD ± 7; range 16–73, median 27) and on average, a caregiver had 3 children. The mean age of children was 26 months (SD ± 14; range 1–53) and the study child was, on average, the third with regard to birth order in the family.

**Table 1:** Socio-demographic characteristics of study participants

Background characteristics	Senti	Mgona	Ntandire	Total
	*N* = 110 (%)	*N* = 188 (%)	*N* = 245 (%)	*N* = 543 (%)
Sex				
Male	3 (2.7%)	4 (2.1%)	2 (0.8%)	9 (1.7%)
Female	107 (97.3%)	184 (97.9%)	243 (99.2%)	534 (98.3%)
Age of caregivers (years)				
16–25	48 (43.6%)	81 (43.1%)	103 (42%)	232 (42.7%)
26–30	27 (24.5%)	54 (28.7%)	77 (31.4%)	158 (29.1%)
≥ 31	35 (31.8%)	53 (28.2%)	65 (26.5%)	153 (28.2%)
Education level of caregivers				
No education / Junior Primary	23 (20.9%)	56(29.8%)	47 (19.2%)	126 (23.2%)
Senior Primary	47 (42.7%)	90 (47.9%)	118 (48.2%)	255 (47%)
Secondary and above	40 (36.4%)	42 (22.3%)	80 (32.7%)	162 (29.8%)
Age of children (months)				
1–11	18(16.4%)	41 (21.8%)	55 (22.4%)	114 (21%)
12–23	32 (29.1%)	42 (22.3%)	51 (20.8%)	125 (23%)
24–36	40 (36.4%)	55 (29.3%)	67 (27.3%)	162 (29.8%)
37–53	20 (18.2%)	50 (25.8%)	72 (29.4%)	142 (25.9%)
Autonomy of caregivers				
Low autonomy	45 (40.9%)	67(35.6%)	98 (40%)	210 (38.7%)
High autonomy	65 (59.1%)	121 (64.4%)	147 (60%)	333 (61.3%)
Social Capital				
Low	48 (43.6%)	78 (41.5%)	94 (38.4%)	219 (40.5%)
High	62 (56.4%)	110 (58.5%)	151 (61.6%)	323(59.5%)
Marital Status				
Single/Divorced/Widow	15 (3.6%)	17 (9.1%)	21 (8.6%)	53 (9.8%)
Married	95 (86.4%)	171 (90.9%)	224 (91.4%)	490 (90.2%)
Maternal ethnicity				
Chewa	44 (40%)	53 (28.2%)	76 (31%)	173 (31.9%)
Ngoni	23 (20.9%)	53 (28.2%)	74 (30.2%)	150 (27.6%)
Lomwe	23 (20.9%)	37 (19.7%)	38 (15.5%)	98 (18.%)
Others	20 (18.2%)	45 (23.9%)	57 (23.3%)	122 (22.5%)

### Childhood morbidity

Of the 543 caregivers who responded to the questionnaire, the majority, 461 (85%) reported a childhood illness episode in the three months preceding the date of interview. Patterns of morbidity indicate that fever was the most prevalent (77%), followed by diarrhoea (42%) and Acute Respiratory Infection (ARI) symptoms characterised by coughing and fast breathing (36%). Caregivers from Senti reported a slightly higher burden of any of the three illness symptoms of interest (84%) among their children than their counterparts in Mgona (77%) and Ntandire (79%). Co-morbidity among symptoms of interest (fever, diarrhoea and ARI) was also noted among 59 (13%) for fever, diarrhoea and ARI symptom combination; 110 (24%) for fever and diarrhoea; and 127 (28%) for fever and ARI symptoms.

### Care-seeking and timely care-seeking from a biomedical health provider

Healthcare from a biomedical provider was sought by 61% (281/461) of the children with reported childhood illness. Public health facilities were the most utilised with an aggregate of 86% (health centre, 69%; central hospital, 11% and district hospital, 6%) with a small proportion, 7% utilising private health facilities. Provider switching which entails seeking care from two or more health providers for the same illness episode was reported by only 10% of caregivers who sought care.

Only 53% (149/281) of caregivers who sought care, did so in a timely manner. Main reasons provided for delay in care-seeking included: inability to quickly recognize symptoms, perception that childhood illness was not severe, and lack of finances and transport to support seeking healthcare from a health facility.

### Factors associated with healthcare seeking and timely care-seeking from a biomedical health provider

Table 2 presents results of two multivariate logistic regression models of factors associated with care-seeking and timely care-seeking from a biomedical health provider. Factors included in the initial analyses using univariate regression models for both care-seeking and timely care-seeking from a biomedical health provider constituted: care giver factors (age; tribe; slum residence; parity; education level; autonomy and social capital); child factors (age and birth order); child illness factors (child illness symptoms such as fever, diarrhoea, ARI; perceived severity; and home management of illness); and household factors (distance to nearest most used health facility, paternal education, and household wealth). Factors with *P values ≤ 0.10* in initial univariate logistic regression models were retained in the final multivariate model. However, education of the care giver and age of the child variables were treated as potential confounders hence were retained in final multivariate models for both care-seeking and timely care-seeking regardless of the result in in initial model.

**Table 2:** Multivariate logistic regression results of factors associated with care-seeking and timely care-seeking from a biomedical health provider

Respondent characteristics	Care seeking		Timely care seeking	
	*n* = 281 (%)	Adjusted OR (95% CI)	*n* = 149 (%)	Adjusted OR (95% CI)
Slum name				
Senti	68 (71.6%)	1.0	40 (58.8%)	1.0
Mgona	97 (60.6%)	0.64 (0.36–1.14)	61 (62.9%)	0.92 (0.45–1.85)
Ntandire	116 (56.3%)	0.56 (0.32–0.98)*	48 (41.4%)	0.59 (0.29–1.19)
Caregiver’s education				
No education/adult literacy	57(54.8%)	1.0	30 (52.6%)	1.0
Primary	141 (64.4%)	1.82 (0.84–3.91)	75 (53.2%)	1.21 (0.41–3.54)
Secondary +	83 (60.1%)	1.55 (0.67–3.61)	44 (53%)	1.17 (0.38–3.59)
Age of caregiver				
16–25	129 64.5%)	1.0	69 (53.5%	NA
26–30	86 (65.2%)	1.20 (0.71–2.02)	44 (51.2%)	
≥ 31	66 (51.2%)	0.85 (0.44–1.64)	36 (54.5%)	
Age of children (in months)				
1–11	64 (71.9%)	1.0	35 (54.7%)	1.O
12–23	73 (62.4%)	0.60 (0.32–1.14)	42 (57.5%)	0.96 (0.46–2.00)
24–36	82 (59.9%)	0.68 (0.37–1.27)	39 (47.6%)	0.69 (0.34–1.41)
37–53	62 (52.5%)	0.54 (0.30–0.99)*	33 (53.2%)	1.02 (0.48–2.15)
Knowledge of danger signs				
No/little knowledge	202 (58.6%)	1.0	112 (55.4%)	1.0
Knowledgeable	79 (68.1%)	1.52 (0.94–2.47)	37 (46.8%)	0.57 (0.33–0.99)*
Home management of illness undertaken				
No	145 (68.4%)	1.0	92 (63.4%)	1.0
Yes	136 (54.6%)	0.54 (0.36–0.81)**	57 (41.9%)	0.44 (0.27–0.74)**
Distance (walking minutes)				
≤ 30 Minutes	102 (60.4%)		61 (59.8%)	1.0
31–60 Minutes	106 (61.6%)	NA	63 (59.4%)	1.12 (0.62–2.04)
> 60 Minutes	73 (60.8%)		25 (34.2%)	0.53 (0.25–1.15)
Perceived illness severity				
Minor				
Moderate	45 (51.7%)	1.0	26 (57.8%)	NA
Severe	126 (56.8%) 110 (72.4%)	1.17 (0.69–2.00) 2.40 (1.34–4.30)**	68 (54%) 55 (50.%)	
Fever				
No	54 (50.9%)	1.0	26 (48.1%)	NA
Yes	227 (63.9%)	1.77 (1.10–2.86)*	123 (54.2%)	
Diarrhoea				
No	153 (57.3%)	1.0	76 (49.7%)	NA
Yes	128 (66%)	1.28 (0.84–1.97)	73 (57%)	
ARI Symptoms				
No	128 (54.7%)	1.0	66 (51.6%)	NA
Yes	83 (62.9%)	1.40 (0.90–2.17)	39 (47%)	

Note: * = *p* < 0.05, ** = *p* < 0.01, *** = *p* < 0.001;

1.0 represents a reference category

NA entails not applicable and that it was not included in the multivariate logistic regression model

Age of the child influenced care-seeking in that healthcare was less likely sought for the oldest age group of children (37–53 months) compared to their younger counterparts aged 1 to 11 months (*AOR = 0.54; 95% CI: 0.30–0.99)*. Provision of home management to a sick child was negatively associated with care-seeking (*AOR = 0.54; 95% CI: 0.36–0.81).* Residence in Ntandire was also found to be negatively associated with care-seeking from a biomedical health provider (*AOR = 0.54; 95% CI: 0.36–0.81).* Child illness severity of a higher order predicted care seeking (*AOR = 2.40; 95% CI: 1.34–4.30)* and a child with fever symptoms was twice as likely to be taken to a health facility than the one who did not exhibit fever as a symptom of illness (*AOR = 1.77; 95% CI: 1.10–2.86).*

With regard to timely care-seeking, caregivers who reported to have undertaken home management for their ill children were less likely to seek care timely (*AOR = 0.44; 95% CI: 0.27–0.74).* Likewise, caregivers with good knowledge of child danger signs were less likely to seek healthcare from a biomedical health provider in a timely manner (*AOR = 0.57; 95% CI: 0.33–0.99).* Overall, majority of care givers, 399 (73.6%) had no to very limited knowledge of child danger signs. Caregiver’s autonomy, social capital; and household wealth did not have any significant association with either healthcare seeking or timely care-seeking from a biomedical health provider at 0.1 significance level and were all excluded in the final logistic regression model.

## Discussion

This study has found that 61% of caregivers sought healthcare albeit 53% of these sought healthcare late. Healthcare was more likely to be sought: for younger than older under-five children, when illness was perceived to be severe, and when the presenting symptom was fever. Home management of childhood illness was negatively associated with care-seeking and timely care-seeking. Caregivers with good knowledge of child danger signs were also less likely to seek care timely.

The rate of healthcare seeking, at 61% is in general, relatively better than that reported in some child healthcare seeking studies [45–47] but still reflects low uptake of interventions. As Pokhrel argued, there is need for significant uptake if optimal benefits of cost-effective interventions are to be realised [48]. More efforts are therefore required to promote child health service utilisation in urban slums in Malawi. Implementation of integrated Community Case Management (iCCM) of common childhood illnesses has yielded positive results in increasing access to and improving quality of essential child health services in Malawi and elsewhere [49, 50].

The iCCM entails provision of basic treatment (curative) services for common childhood symptoms of mild to moderate severity by a trained lay cadre of community health workers with view of reducing geographical barrier and fostering prompt treatment [Personal communication with official from Ministry of Health]. In Malawi, iCCM has only been implemented in rural areas (especially hard to reach areas) using Health Surveillance Assistants – a cadre of lay community health workers. It may be worthwhile to consider implementing iCCM even in urban slums granted our study findings that healthcare seeking, although relatively high, is still suboptimal and that care is sought late by almost half of those that seek it. In the context of urban health, iCCM in urban slums will reduce congestion at urban health centres effectively lessening the burden of long waiting times, and potentially contributing to more time spent on examining children with severe illness at a health facility. Indeed, long waiting times and suboptimal examination of the sick child by health workers have been shown to have negative effect on preference for health facility in the event of childhood illness [51–53]. It entails that implementing iCCM may arguably also have a positive effect on overall use of biomedical health services at facility level.

Whilst we acknowledge the definition of prompt care-seeking being care sought within 24 h period as cited hitherto, based on Heads of States commitment on Roll Back Malaria, similar to Rutebemberwa et al. (2010), we used definition of ‘next day’ from onset of symptoms to approximate the 24-h period. This approach was deemed to be more practical and contextually relevant for eliciting time in our study granted low education levels and that people hardly use clocks/watches to undertake time (in this case, hours) estimations. Our experience in this study therefore renders support to a call for more reliable methods of retrospective time estimation in surveys conducted in developing country contexts, especially among participants of low literacy.

Consistent with some similar studies [36, 54, 55], our results indicate that caregivers were more likely to seek healthcare from a biomedical health provider for younger children. The greater dependence of younger children on their caregivers may entail more observation and subsequently response of seeking care from a health facility in the event of illness. Moreover, caregivers may be aware of faster disease progression in younger children with whom they are in close contact relative to older ones hence more likely to watchfully wait to seek healthcare for older children.

Our study has also demonstrated that undertaking home management of childhood illness negatively influenced both care-seeking and timely care-seeking from a health facility. This may be explained by the possibility that in some cases, home management may have led to childhood symptoms subsiding, effectively eliminating the need for care-seeking from a health provider. Additionally, caregivers may have been watchfully waiting for the effect of their home remedies hence delayed in seeking care. Implementation of community IMCI in Malawi and elsewhere provides for educating caregivers on home management of some less severe cases of childhood illnesses [56]. The fact that about 55% of caregivers who had undertaken home management for their child’s illness subsequently sought healthcare, and among them 42% sought it late (See Table 2) suggests that prompt care-seeking from a biomedical health provider was the appropriate response in the first place. In view of these findings, it is imperative that appropriate health education is undertaken so that home management is not seen as an end in itself and that it does not compromise prompt care-seeking which is essential for positive child health outcomes from illness episodes.

Additionally, ensuring that home management is conducted correctly represents an important area of focus but one which was beyond the scope of our study. Evidence pertaining to appropriateness of home management undertaken for various childhood illnesses including determinants of home management in the context of IMCI remains sparse in Malawi and among caregivers in urban slums, in particular. Specific evidence in this area is therefore warranted.

Unsurprisingly, illness severity influenced healthcare seeking as the odds of seeking care from a biomedical health provider were more than twice for childhood illness perceived to be more severe relative to that perceived to be of minor severity. This renders support to similar findings in studies conducted in Malawi [54, 57], Kenya [36], Ethiopia [58] and Uganda [42] albeit they contrast with findings from a study in rural Ethiopia where perceived severity did not necessarily trigger care-seeking for neonates [59]. Indeed, as childhood condition progresses in severity, it stretches beyond the scope that caregivers may attempt home treatment hence seeking help from a health facility. Nonetheless the use of variables denoting severity of illness as perceived by caregivers have previously been contested [36] mainly on the basis that they rely on caregivers to undertake an implicit severity rating, which may require technical expertise especially if measurements are to be standard and easily comparable. While this is a valid argument and calls for robust methods of eliciting such information, it is noteworthy that decisions to seek care are undertaken by the same caregivers and hence their very perception of illness severity probably provides good indications of decision making dynamics.

In our study, having fever predicted care-seeking but ARI symptoms and diarrhoea did not. Since, by classification, ARI symptoms are characterised by coughing and fast breathing, the expectation was that this combination would have been considered serious enough to warrant care-seeking from a health provider. Children with ARI symptoms need prompt antibiotic treatment available from a health facility, hence this finding presents a public health challenge for child health in urban slums that may be arising from lack of knowledge on the seriousness of ARI symptoms. Similar results, albeit for coughing symptoms were found in a study in the Nairobi slums in Kenya. Rationalising this finding, the authors posited that the fact that respiratory infections were so common in urban slum setting of their study, caregivers may have thought illness with respiratory infection symptoms were like any other experienced many times before hence did not bother seeking healthcare from a facility [36]. Indeed, even though pneumonia is one of the commonest causes of childhood morbidity and mortality in Malawi, care-seeking has been low in some years, albeit the recent demographic and health survey reports good healthcare seeking rate of 83.5% for ARI [6].

While distance to health facility has been shown to be a significant predictor of care-seeking, timely care-seeking and risk of child mortality in previous studies elsewhere [10, 42, 60, 61], our results did not demonstrate the inverse relationship common in many studies. This finding may relate to an observation by the Knowledge Network on Urban Settings (KNUS) for the World Health Organisation’s Commission on Social Determinants of Health that within urban areas, it is cost and not proximity to health facilities that may be key to accessing healthcare in urban areas [22]. Moreover, some discrete choice experiment studies have also demonstrated that distance to health facility may not be a very significant determinant factor influencing choice of health facility or care-seeking as people are willing to travel longer if any of the other significant health facility considerations are present [51, 62].

In our study, household wealth did not predict care-seeking or timely care-seeking from health facilities. It is probable that the fact that a significant majority of caregivers utilized public health facilities which provide free services at the point of use in Malawi, entailed that household wealth may not have had a significant effect in care-seeking dynamics. Relatedly, our discrete choice experiment study responded to by a subset of participants used in this study revealed that cost of healthcare negatively influenced the choice of a health facility in the event of childhood illness, effectively intimating caregivers’ aversion with cost of healthcare [51].

Similarly, autonomy and social capital of caregivers did not significantly predict care-seeking or timely care-seeking from biomedical health providers. These results contrast with those found elsewhere that demonstrated autonomy [63, 64] and social capital [10, 65] to be significant predictors of care-seeking, timely care-seeking and protective against child mortality [43].

Knowledge of child danger signs neither determined care-seeking nor timely care-seeking in our study. This contrasts with other studies that have demonstrated that knowledge of danger signs or symptom recognition is a critical predictor of care-seeking for child health services [42, 66]. Indeed, promotion of health education on child danger signs to caregivers within the Community-IMCI strategy is on the premise that knowledge of child danger signs will promote uptake of child health services. However, other research findings have equally been divergent in this regard [67, 68] and seemingly render support to the lack of significant association noted in this study. For example, in a study conducted in Egypt, mothers were provided with brief descriptions of childhood illnesses and asked how they would respond in each case. Results from this were compared with their actual responses to repeated subsequent episodes of ARI. In spite of mothers’ recognition of child danger signs, their knowledge did not lead to taking appropriate action of seeking healthcare from the health facility [42, 67]. This portrays the complexity of healthcare seeking and the need to consider factors beyond symptom recognition and/or knowledge of child danger signs.

Some limitations are acknowledged for our study. We relied on self-reported information hence there is potential for reporting bias. There was also potential for recall bias considering the long recall period. However, we undertook verification of reported child morbidity and care-seeking information with what is documented in the child health passports – a health booklet that keeps record of health information including symptom description, frequency and duration, at individual client level. This arguably reduced reporting and recall bias. The cross-section nature of the study limited opportunity to observe any seasonal or time variations in both disease patterns and corresponding healthcare-seeking dynamics, both of which could have contributed to a deeper understanding of the phenomenon under study. The study’s strengths relate to: a rigorous participant selection process which included an initial census to enumerate study participants; focusing on an important group of the population (i.e. residents of urban slums) which is understudied albeit increasing in size due to rapid urbanization, slow economic growth and the so-called urbanization of poverty.

While our study focused on a general issue pertaining to healthcare seeking for under-five child health services, generalization of findings may be comfortably undertaken only in similar contexts of urban slums. Furthermore, some findings may need to be interpreted with caution, such as the lack of association between education of caregiver to care-seeking and timely care-seeking, given established evidence in this regard.

## Conclusion

In conclusion, the findings of this study show that healthcare seeking from a biomedical health provider among caregivers of under-five children in urban slums remains suboptimal and mostly sought untimely. Factors related to illness of the child (severity, type of symptom, and whether illness is initially managed at home); the child (age of child) and geographical environment (slum residence) influence care-seeking decisions.

The results of the baseline study point to various implications for policy and practice. There is need to focus on child health programming as delivered to residents in urban slums especially with regard to interface between facility based IMCI and the community component in so far as strategies to promote child healthcare seeking and the actual utilisation of health services are concerned. The concept of Community Case Management, while at present considered more appropriate for rural areas who are relatively farther away from health facilities, might as well be appropriate for caregivers in urban slums in Malawi. While symptom recognition, knowledge of danger signs and home management of common childhood illness are important programmatically, health education is imperative so that they do not hinder appropriate care-seeking.

Disaggregated analysis of urban health data is essential for epidemiological mapping that should inform context specific strategies of delivering child health interventions. The extent to which child health and survival efforts are promoted in urban Malawi therefore depends on acknowledgement that residents in urban slums may not enjoy a health advantage as aggregate urban health indicators suggest. Furthermore, the cognizance that the urban context in itself is a determinant of health hence the need for an urban health agenda that responds to the differential needs of different urban population groups should warrant a deeper look in urban child health research, policy and practice.

## Data Availability

The datasets used and/or analysed during the current study are available from the corresponding author on reasonable request.

## Abbreviations

C-IMCI: Community Integrated Management of Childhood Illnesses
CSDH: Commission on Social Determinants of Health
DHS: Demographic Health Survey
iCCM: Integrated Community Case Management
IMCI: Integrated Management of Childhood Illnesses
KNUS: Knowledge Network on Urban Settings
MDG: Millennium Development Goals
NHSRC: National Health Sciences Research Ethics Committee
SDG: Sustainable Development Goals
